# Bis[4-bromo-2-(ethyl­imino­meth­yl)phenolato-κ^2^ *N*,*O*]nickel(II)

**DOI:** 10.1107/S1600536811020885

**Published:** 2011-06-04

**Authors:** Fu Li Chen, Shu Hua Zhang, Jing Jing Guo, Yi Dong Zhang, Chao Feng

**Affiliations:** a College of Chemistry and Bioengineering, Guilin University of Technology, Key Laboratory of Non-ferrous Metal Materials and Processing Technology, Ministry of Education, Guilin 541004, People’s Republic of China

## Abstract

In the title complex, [Ni(C_9_H_9_BrNO)_2_], the Ni^II^ ion lies on an inversion centre and is coordinated in a slightly distorted square-planar geometry by two N atoms and two O atoms from two symmetry-related bidentate 4-bromo-2-(ethyl­imino­meth­yl)phenolate ligands. The complex forms a one-dimensional chain in the crystal structure through short C—H⋯Br contacts (H⋯Br = 3.009 Å).

## Related literature

For background to Schiff base compounds, see: Gupta & Sutar (2008 ▶); Zhang *et al.* (2008 ▶, 2009 ▶); Zhang & Feng (2010 ▶); Ge *et al.* (2011 ▶). For Schiff base coordination models, see: Nakagima *et al.* (1989 ▶); Zhang *et al.* (2007 ▶).
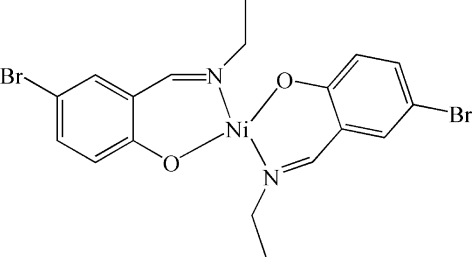


## Experimental

### 

#### Crystal data

[Ni(C_9_H_9_BrNO)_2_]
*M* *_r_* = 512.83
Monoclinic, 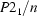

*a* = 13.456 (6) Å
*b* = 4.803 (2) Å
*c* = 14.743 (6) Å
β = 102.157 (8)°
*V* = 931.4 (7) Å^3^
*Z* = 2
Mo *K*α radiation
μ = 5.35 mm^−1^
*T* = 293 K
0.15 × 0.12 × 0.11 mm

#### Data collection

Bruker SMART CCD area-detector diffractometer
Absorption correction: multi-scan (*SADABS*; Bruker, 2004 ▶) *T*_min_ = 0.465, *T*_max_ = 0.558
4567 measured reflections
1651 independent reflections
995 reflections with *I* > 2σ(*I*)
*R*_int_ = 0.164

#### Refinement

*R*[*F* ^2^ > 2σ(*F* ^2^)] = 0.057
*wR*(*F* ^2^) = 0.142
*S* = 1.03
1651 reflections
116 parameters
H-atom parameters constrained
Δρ_max_ = 0.79 e Å^−3^
Δρ_min_ = −0.52 e Å^−3^

### 

Data collection: *SMART* (Bruker, 2004 ▶); cell refinement: *SAINT* (Bruker, 2004 ▶); data reduction: *SAINT*; program(s) used to solve structure: *SHELXS97* (Sheldrick, 2008 ▶); program(s) used to refine structure: *SHELXL97* (Sheldrick, 2008 ▶); molecular graphics: *SHELXTL* (Sheldrick, 2008 ▶); software used to prepare material for publication: *SHELXL97*.

## Supplementary Material

Crystal structure: contains datablock(s) I, global. DOI: 10.1107/S1600536811020885/bh2357sup1.cif

Structure factors: contains datablock(s) I. DOI: 10.1107/S1600536811020885/bh2357Isup2.hkl

Additional supplementary materials:  crystallographic information; 3D view; checkCIF report

## Figures and Tables

**Fig. 1.**
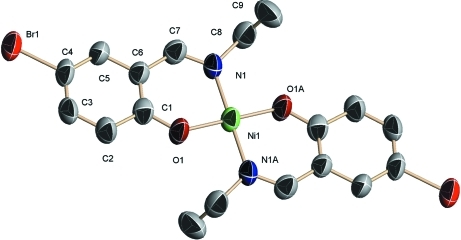
The molecular structure of the title complex, showing 30% probability displacement ellipsoids. H atoms were omitted.

**Fig. 2.**
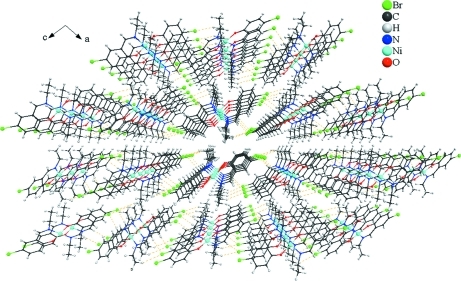
Packing drawing of the title compound.

## supplementary crystallographic information

### Comment

Schiff base complexes have been studied for many years (Gupta & Sutar, 2008; Zhang *et al.*, 2008, 2009; Zhang & Feng, 2010; Ge *et al.*, 2011) and produced increasing interest because of their anticancer, antiviral, catalytic and fluorescent properties. Most model studies of metal complexes of Schiff base ligands containing salicylaldehyde and amino acids have focused on the binding mode of these ligands (Nakagima *et al.*, 1989; Zhang *et al.*, 2007). The crystal structures of the complexes obtained demonstrate that the Schiff base ligands act in a bidentate, tridentate, tetradentate or pentadentate mode, coordinating through the phenolate O, imine N and carboxylate O atoms. Our research group is interested in bidentate Schiff bases derived from 5-bromo-2-hydroxy-benzaldehyde and ethylamine.

In the title complex, the Ni^II^ ion lies on a centre of inversion and is coordinated by two O and two N atoms from two bidentate 5-bromo-*N*-ethylsalicylaldimino ligands, forming a slightly distorted square-planar geometry (Fig. 1). The compound further form a one-dimensional crystal structure (Fig. 2) through C—H···Br contacts (C9···Br1^i^ = 3.871 (1) Å, H9···Br1 = 3.009 Å, symmetry code: (i) -*x*, -*y*, 1 - *z*).

### Experimental

To a solution of 5-bromo-2-hydroxy-benzaldehyde (0.181 g, 1.0 mmol), ethylamine (0.044 g, 1 mmol), and sodium hydroxide (0.040 g, 1 mmol) in 20 ml absolute methanol was added slowly a solution of nickel nitrate hexahydrate (0.145 g, 0.5 mmol) in methanol. The mixture was stirred for 3 h at room temperature to give a green solution, which was filtered and the filtrate was left to stand at room temperature. Green block crystals suitable for X-ray diffraction were obtained by slow evaporation. yield: 84.6% (based on Ni). Elemental analysis, calculated: C 42.12, H 3.57, N 5.48%; Found: C 42.15, H 3.54, N 5.46%.

### Refinement

H atoms were positioned geometrically and refined with a riding model, with distances 0.96 (CH_3_), 0.97 (CH_2_) or 0.93 Å (aromatic CH), and with *U*_iso_(H) = 1.2 *U*_eq_(carrier C) or *U*_iso_(H) = 1.5 *U*_eq_(CH_3_).

### Crystal data

**Table:**

[Ni(C_9_H_9_BrNO)_2_]	*F*(000) = 508
*M**_r_* = 512.83	*D*_x_ = 1.829 Mg m^−^^3^
Monoclinic, *P*2_1_/*n*	Mo *K*α radiation, λ = 0.71073 Å
Hall symbol: -P 2yn	Cell parameters from 1651 reflections
*a* = 13.456 (6) Å	θ = 2.3–25.1°
*b* = 4.803 (2) Å	µ = 5.35 mm^−^^1^
*c* = 14.743 (6) Å	*T* = 293 K
β = 102.157 (8)°	Block, green
*V* = 931.4 (7) Å^3^	0.15 × 0.12 × 0.11 mm
*Z* = 2	

### Data collection

**Table:**

Bruker SMART CCD area-detector diffractometer	1651 independent reflections
Radiation source: fine-focus sealed tube	995 reflections with *I* > 2σ(*I*)
graphite	*R*_int_ = 0.164
φ and ω scans	θ_max_ = 25.1°, θ_min_ = 2.3°
Absorption correction: multi-scan (*SADABS*; Bruker, 2004)	*h* = −15→16
*T*_min_ = 0.465, *T*_max_ = 0.558	*k* = −5→5
4567 measured reflections	*l* = −17→14

### Refinement

**Table:**

Refinement on *F*^2^	Primary atom site location: structure-invariant direct methods
Least-squares matrix: full	Secondary atom site location: difference Fourier map
*R*[*F*^2^ > 2σ(*F*^2^)] = 0.057	Hydrogen site location: inferred from neighbouring sites
*wR*(*F*^2^) = 0.142	H-atom parameters constrained
*S* = 1.03	*w* = 1/[σ^2^(*F*_o_^2^) + (0.0386*P*)^2^] where *P* = (*F*_o_^2^ + 2*F*_c_^2^)/3
1651 reflections	(Δ/σ)_max_ < 0.001
116 parameters	Δρ_max_ = 0.79 e Å^−^^3^
0 restraints	Δρ_min_ = −0.52 e Å^−^^3^
0 constraints	

### Fractional atomic coordinates and isotropic or equivalent isotropic displacement parameters (Å^2^)

**Table:**

	*x*	*y*	*z*	*U*_iso_*/*U*_eq_	
Br1	0.14681 (6)	0.5969 (2)	0.56509 (5)	0.0835 (4)	
C1	0.0224 (5)	0.9743 (16)	0.8127 (5)	0.0595 (18)	
C2	−0.0114 (6)	1.0740 (16)	0.7195 (5)	0.069 (2)	
H2	−0.0590	1.2170	0.7075	0.083*	
C3	0.0255 (5)	0.9609 (18)	0.6489 (4)	0.067 (2)	
H3	0.0025	1.0257	0.5887	0.080*	
C4	0.0964 (5)	0.7519 (18)	0.6657 (4)	0.065 (2)	
C5	0.1313 (5)	0.6526 (17)	0.7529 (4)	0.064 (2)	
H5	0.1806	0.5139	0.7635	0.077*	
C6	0.0921 (5)	0.7616 (15)	0.8278 (4)	0.0556 (17)	
C7	0.1305 (5)	0.6525 (16)	0.9196 (5)	0.0638 (19)	
H7	0.1794	0.5130	0.9257	0.077*	
C8	0.1627 (6)	0.5937 (18)	1.0814 (5)	0.080 (3)	
H8A	0.1164	0.5395	1.1206	0.096*	
H8B	0.1951	0.4265	1.0646	0.096*	
C9	0.2412 (6)	0.783 (2)	1.1336 (6)	0.100 (3)	
H9A	0.2914	0.8198	1.0976	0.150*	
H9B	0.2730	0.6973	1.1913	0.150*	
H9C	0.2100	0.9548	1.1459	0.150*	
N1	0.1026 (4)	0.7322 (12)	0.9936 (3)	0.0560 (15)	
Ni1	0.0000	1.0000	1.0000	0.0544 (4)	
O1	−0.0152 (4)	1.0925 (11)	0.8785 (3)	0.0712 (15)	

### Atomic displacement parameters (Å^2^)

**Table:**

	*U*^11^	*U*^22^	*U*^33^	*U*^12^	*U*^13^	*U*^23^
Br1	0.0908 (6)	0.1153 (9)	0.0524 (5)	−0.0021 (5)	0.0331 (4)	−0.0090 (4)
C1	0.069 (4)	0.064 (5)	0.049 (4)	−0.013 (4)	0.021 (3)	0.003 (3)
C2	0.086 (5)	0.078 (6)	0.046 (4)	0.012 (4)	0.017 (4)	0.013 (4)
C3	0.075 (5)	0.090 (6)	0.038 (4)	−0.002 (5)	0.017 (3)	0.011 (4)
C4	0.070 (4)	0.089 (6)	0.043 (4)	−0.017 (4)	0.026 (3)	−0.005 (4)
C5	0.067 (5)	0.081 (6)	0.050 (4)	0.011 (4)	0.022 (3)	−0.001 (4)
C6	0.060 (4)	0.061 (5)	0.049 (4)	0.001 (4)	0.019 (3)	0.001 (3)
C7	0.075 (5)	0.060 (5)	0.062 (5)	0.012 (4)	0.026 (4)	0.008 (4)
C8	0.110 (6)	0.078 (6)	0.059 (5)	0.040 (5)	0.033 (5)	0.024 (4)
C9	0.087 (6)	0.141 (9)	0.066 (5)	0.012 (6)	0.001 (5)	0.029 (6)
N1	0.071 (4)	0.061 (4)	0.038 (3)	0.000 (3)	0.018 (3)	0.004 (3)
Ni1	0.0691 (8)	0.0562 (8)	0.0417 (7)	0.0038 (6)	0.0205 (5)	0.0068 (6)
O1	0.093 (4)	0.083 (4)	0.044 (3)	0.027 (3)	0.030 (2)	0.013 (2)

### Geometric parameters (Å, °)

**Table:**

Br1—C4	1.907 (7)	C7—H7	0.9300
C1—O1	1.314 (9)	C8—C9	1.481 (12)
C1—C6	1.373 (10)	C8—N1	1.527 (8)
C1—C2	1.435 (10)	C8—H8A	0.9700
C2—C3	1.358 (10)	C8—H8B	0.9700
C2—H2	0.9300	C9—H9A	0.9600
C3—C4	1.371 (10)	C9—H9B	0.9600
C3—H3	0.9300	C9—H9C	0.9600
C4—C5	1.359 (9)	N1—Ni1	1.904 (6)
C5—C6	1.420 (9)	Ni1—O1^i^	1.815 (4)
C5—H5	0.9300	Ni1—O1	1.815 (4)
C6—C7	1.442 (9)	Ni1—N1^i^	1.904 (6)
C7—N1	1.284 (8)		
			
O1—C1—C6	124.0 (6)	C9—C8—H8A	109.3
O1—C1—C2	117.9 (7)	N1—C8—H8A	109.3
C6—C1—C2	118.1 (7)	C9—C8—H8B	109.3
C3—C2—C1	120.5 (7)	N1—C8—H8B	109.3
C3—C2—H2	119.8	H8A—C8—H8B	108.0
C1—C2—H2	119.8	C8—C9—H9A	109.5
C2—C3—C4	120.5 (6)	C8—C9—H9B	109.5
C2—C3—H3	119.8	H9A—C9—H9B	109.5
C4—C3—H3	119.8	C8—C9—H9C	109.5
C5—C4—C3	121.1 (7)	H9A—C9—H9C	109.5
C5—C4—Br1	119.4 (6)	H9B—C9—H9C	109.5
C3—C4—Br1	119.6 (5)	C7—N1—C8	113.2 (6)
C4—C5—C6	119.7 (7)	C7—N1—Ni1	126.0 (5)
C4—C5—H5	120.2	C8—N1—Ni1	120.8 (4)
C6—C5—H5	120.2	O1^i^—Ni1—O1	180.000 (2)
C1—C6—C5	120.1 (6)	O1^i^—Ni1—N1^i^	92.8 (2)
C1—C6—C7	121.3 (6)	O1—Ni1—N1^i^	87.2 (2)
C5—C6—C7	118.5 (6)	O1^i^—Ni1—N1	87.2 (2)
N1—C7—C6	125.3 (7)	O1—Ni1—N1	92.8 (2)
N1—C7—H7	117.3	N1^i^—Ni1—N1	180.0 (3)
C6—C7—H7	117.3	C1—O1—Ni1	129.9 (5)
C9—C8—N1	111.4 (7)		

Symmetry codes: (i) −*x*, −*y*+2, −*z*+2.
